# Green Synthesis of Silver Nanoparticles from the Extracts of Fruit Peel of *Citrus tangerina*, *Citrus sinensis*, and *Citrus limon* for Antibacterial Activities

**DOI:** 10.1155/2021/6695734

**Published:** 2021-02-02

**Authors:** Moira Carmalita Dharsika Niluxsshun, Koneswaran Masilamani, Umaramani Mathiventhan

**Affiliations:** ^1^Department of Chemistry, Faculty of Science, Eastern University, Vantharumoolai, Chenkalady 30350, Sri Lanka; ^2^Department of Botany, Faculty of Science, Eastern University, Vantharumoolai, Chenkalady 30350, Sri Lanka

## Abstract

Wide application of nanoparticles motivates the need for synthesising them. Here, a nontoxic, eco-friendly, and cost-effective method has been established for the synthesis of silver nanoparticles using extracts of lemon peel (*Citrus limon*), green orange peel (*Citrus sinensis*), and orange peel (*Citrus tangerina*). The synthesised nanoparticles have been characterised using UV-visible absorptionspectroscopy, Fourier transform infrared spectroscopy, and transmission electron microscopy (TEM). The UV-visible absorption spectrum of these synthesised silver nanoparticles shows an absorption peak at around 440 nm. TEM images show different shaped particles with various sizes. Furthermore, the antibacterial activity of silver nanoparticles was appraised by a well-diffusion method and it was observed that the green synthesised silver nanoparticles have an effective antibacterial activity against *Escherichia coli* and *Staphylococcus aureus*. The outcome of this study could be beneficial for nanotechnology-based biomedical applications.

## 1. Introduction

Metal nanoparticles exhibit unique optical and optoelectronic properties in the size range of 1–100 nm, and therefore, they found widespread applications in various fields such catalysis, electronics, optics, environmental, and biotechnology which is an area of constant interest [1–4]. Among these nanomaterials, silver nanoparticles (AgNPs) are commonly used in many applications due to their unique optical properties, relatively high stability, and strong conjugation ability with biomolecules [5–7]. Several methods include chemical, photochemical, electrochemical, and biological methods have been used to synthesise the different size and shape of the nanoparticles to meet the specific demands of different applications [8–12]. Chemical and physical methods are traditionally involved in the synthesis of nanoparticles cause pollution to the environment. Moreover, chemical routes produce many hazardous by-products which also cause problems to the environment. Therefore, there is a need for “Green Synthesis” of nanoparticles that includes a clean, safe, and eco-friendly route for synthesis of nanoparticles and this synthesis route does not involve in high temperature, pressure, energy, and toxic chemicals [13].

Several eco-friendly approaches have been used to synthesise the nanoparticles since they have several advantages such as cost-effectiveness, simplicity, and compatibility for antibacterial and antioxidant activities. Many scientists are focussed on green synthesis of nanoparticles from plant extracts [14, 15]. AgNPs were also synthesised from various plant parts such as roots, seeds, leaves, stems, and flowers for different applications, especially to the biomedical applications [16]. For instance, aqueous extracts of *Cleome viscosa*, *Origanum vulgare*, *Ocimum tenuiflorum*, *Solanum trilobatum*, *Syzygium cumini*, *Impatiens balsamina*, *Lantana camara*, and *Centella asiatica*were used for the synthesis of AgNPs with antibacterial properties [16–19]. Researchers have also employed *Gelidium amansii*, *Enteromorpha compressa*, *Phanerochaete chrysosporium*, *Bacillus brevis*, and *Daucus carota* for the biosynthesis of AgNPs and examined their antimicrobial properties in the past decades [13].

Contamination of wounds by bacteria is a common problem, and therefore, prevention of wound contamination is important. Many medicines are recommended in the standard wound care. However, an effective antimicrobial dressing may help to promote normal wound healing. Many researchers demonstrated that the AgNP enhanced the antibacterial efficacy *in vitro*, and it is proved that AgNP would be a good candidate for antibacterial wound care [20, 21].

Silver nanoparticles are synthesised by the reduction of silver ions to neutral silver atoms. This is achieved by the reduction of silver ions by a reducing agent [22]. The peel of *Citrus* sp. fruits is a rich source of flavanones and many polymethoxylated flavones, which are very rare in other plants, and these materials could be used as reducing agents in the synthesis of AgNPs. In this research, AgNPs were synthesised through a green synthesis method using the extracts from peels of lemon, orange, and green orange. Antimicrobial activity of the synthesised AgNPs against the isolated clinically significant bacterial strains such as *Escherichia coli* and *Staphylococcus aureus* was studied in this work.

## 2. Material and Methods

### 2.1. Materials

Silver nitrate was purchased from Organic Trading (Pvt) Ltd. Lemon (*Citrus limon*) fruits were obtained from the farm of the Department of Agriculture, Eastern University, Sri Lanka (EUSL). Green oranges *(Citrus sinensis)* were plucked from the home garden in Batticaloa, Sri Lanka, and the orange fruits *(Citrus tangerina)* were purchased from a super market in Batticaloa. Fresh grown pure cultures of *Escherichia coli* and *Staphylococcus aureus* were taken from the Department of Microbiology, Faculty of Health Care Sciences, EUSL. All solutions were prepared in distilled water. All glass wares were properly washed with distilled water and dried in oven before use.

### 2.2. Green Synthesis of Silver Nanoparticles

#### 2.2.1. Preparation of Peel Extracts

The lemon, orange, and green orange peels were washed thoroughly with distilled water and cut into small pieces. 8 g of each fruit peels was transferred into 80 ml of distilled water and boiled for 2–5 minutes separately. The extracts obtained were filtered through filter paper, and each filtrate was collected in 250 ml Erlenmeyer flask separately and stored at 4°C for further use [23].

#### 2.2.2. Preparation of Silver Nanoparticles

**Figure 1:**
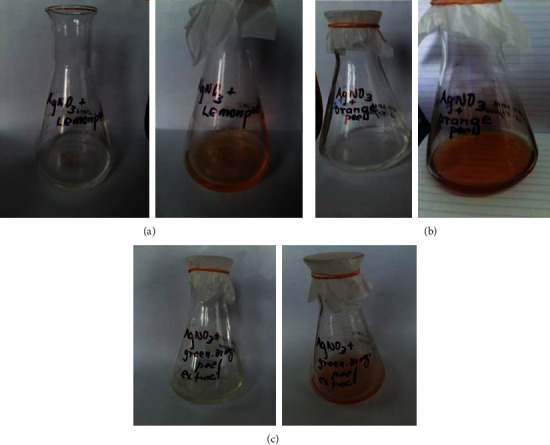
Colour change in the solution after the addition of (a) lemon peel extract, (b) orange peel extract, and (c) green orange peel extracts due to reduction of silver ions.

For the green synthesis of silver nanoparticles, 3 ml of each fruit extract was carefully added to 40 ml of 1 mM aqueous AgNO_3_ solution in 250 ml flask and kept it at room temperature for 5 hours under dark condition. A change in colour from colourless to reddish brown or golden brown of colloidal suspension confirmed the biosynthesis of silver nanoparticles [24] (Figure 1).

### 2.3. Characterisation

The UV-Vis Absorption spectra of synthesised AgNPs were recorded at different time intervals using Biobase D580 series double-beam UV-visible spectrophotometer with the slit width and spectral bandwidth of 1.0 nm. Absorption spectra of several-week-old AgNPs samples were also recorded to check their stability. Fourier Transform Infrared (FT-IR) spectra were recorded in the range 4000–400 cm^−1^ using a Thermo Scientific (Bruker Vertex 80) FT-IR spectrophotometer. The sample was placed on the diamond crystal plate, and the spectrum was recorded using ATR mode. Morphological studies of these nanoparticles were performed using an (JEM2100) HR TEM JEOL Transmission Electron Microscopy (TEM).

AgNPs samples were prepared for TEM imaging by placing a 1.0 *μ*l of AgNPs colloidal suspension on carbon-coated 400 mesh copper grid, allowing the film on the TEM grid to stand for 2 hours and letting the grid dry prior to measurement.

### 2.4. Test Microorganism and Microbial Inoculum

Two human pathogenic bacteria such as *Escherichia coli* and *Staphylococcus aureus* were chosen for this study. Both bacterial strains were developed in the nutrient agar medium at 37°C (the bacteria were grown in the nutrient broth at 37°C and preserved on nutrient agar slants at 4°C).

### 2.5. Antimicrobial Activity

Antibacterial activities of silver nanoparticles synthesised in three different approaches were examined using a standard well-diffusion method against two human pathogenic bacteria such as *Escherichia coli* and *Staphylococcus aureus* [25]. Around 15–20 ml of nutrient agar was poured on glass Petri dishes, and we allowed them to solidify. Agar surface of each plate was streaked by a sterile cotton swab with the respective bacterial strain. An agar plate was punched with a sterile cork borer of 5 mm size. Varying volumes (20, 40, 60, 80, and 100 *μ*l) of solution containing nanoparticles were inoculated in these holes. The plates were allowed to standby for 30 min to 1 hour. The plates were incubated at 37°C for 48–72 hours. Then, the zone of inhibition was measured.

The addition of lemon, orange, and green orange peel extracts to the silver nitrate solution resulted in the change in colour from colourless to reddish brown on incubation for 5 hours at room temperature. A slight colour change was observed within half an hour after the addition of fruit peel extracts and the intensity of the colour increased with time. The colour changes in the solutions were seen due to the surface plasmon vibrations of the silver nanoparticles [26, 27]. Change in colour after the reduction of silver ions (Ag^+^) to silver nanoparticles (Ag^0^) is shown in Figure 1.

## 3. Results and Discussion

### 3.1. UV-Visible Spectroscopy

**Figure 2:**
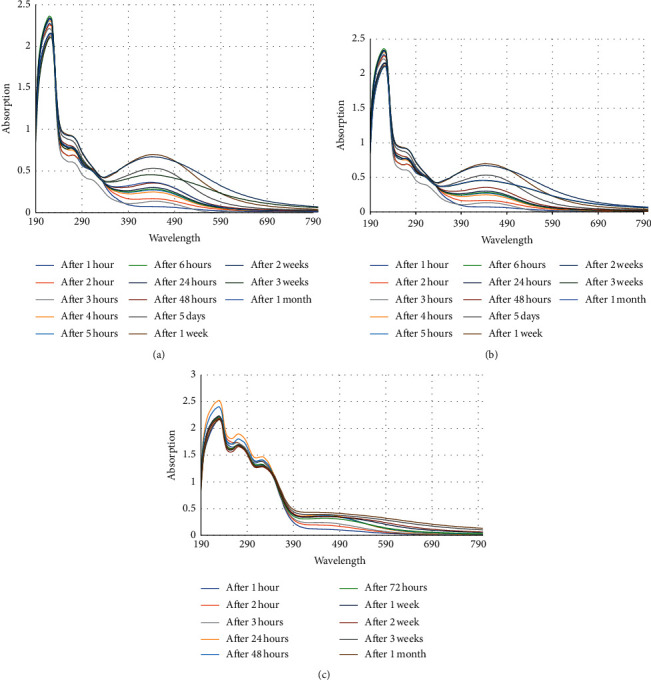
UV-visible spectrum recorded at different time intervals for 1 mM solution of silver nitrate with (a) lemon peel, (b) orange peel, and (c) green orange peel extracts.

UV-visible spectra which were recorded at different time intervals for aqueous solution of silver nitrate with lemon peel extract are shown in Figure 2(a), orange peel extract are shown in Figure 2(b), and green orange peel extract are shown in Figure 2(c). All three samples displayed an optical absorption band peak at around 430–450 nm, typical of absorption for metallic Ag nanoparticles, due to the surface plasmon resonance similar to those reported in the literature [28]. There are slight variations in the values of absorbance between these three extracts which signifies that the changes are due the particle size or shape [29]. Effect of reaction time on the silver nanoparticle synthesis was also studied with UV-Vis absorption spectra, and it is observed that with an increase in time, the peaks become more intense. The increase in intensity is due to increase in the number of nanoparticle development due to reduction reaction between silver ions and biomolecules exist in the aqueous solution. However, after 48 hours of reaction time, intensity of surface plasmon resonance band becomes almost constant, indicating the completion of reaction. However, after 2 weeks, the intensity of the peak started to decrease for lemon and orange peel (Figure 2(a) and 2(b)) and the absorption peak started to shift to higher wavelength for green orange peel (Figure 2(c)). During this process, there were no significant changes observed in the colour of the solutions. This indicates that the stability of silver nanoparticles synthesised using these fruit peel extracts was lasted for about 2–3 weeks after that the particles started to accumulate, which is showed by the shifts in the absorption peaks. Moreover, UV-visible spectral analysis was made to 1 mM silver nitrate solution, plain lemon peel extract, orange peel extract, and green orange peel extract, and it was observed that they did not display any characteristic peaks.

### 3.2. FT-IR Spectroscopy

**Figure 3:**
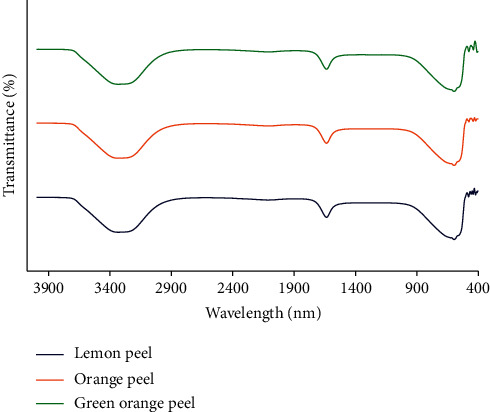
FT-IR spectrum of silver nanoparticles synthesised using lemon peel, orange peel, and green orange peel extracts.

. The fruit peel extracts were used as a reducing agent to synthesise the silver nanoparticles, and therefore, FT-IR spectroscopy was used to confirm this reduction process. On this aspect, the functional groups presence on the surface of the silver nanoparticle were studied using the FT-IR spectroscopy. This result confirmed the possible formation of bio reduction and efficient stabilization of green synthesised silver nanoparticles by using Citrus sp. peel extracts. As shown in Figure 3, all three FT-IR spectra of silver nanoparticles synthesised using extracts of lemon peel, orange peel, and green orange peel are shown in the absorption peaks around 2950–3670, 3331, 2115, 1636, and 597 cm^−1^. A broad absorption band between 2950 and 3670 cm^−1^ is due to the O-H stretching frequency. The band at 1636 cm^−1^ corresponds to C=O stretching of the carbonyl group and aromatic C=C stretching vibration. A weak peak around 2115 cm^−1^ could be assigned to the alkyne group present in phytochemicals of the extracts. The broad peaks around 597 cm^−1^ are related to silver nanoparticle bonding with oxygen from hydroxyl groups. There is no or not much variation among the FT-IR spectrum of the silver nanoparticles synthesised using these three different fruit peel extracts. These IR peaks are mainly attributed to flavanoids and terpenoids excessively present in plant extract which are used as the reducing agents during the synthesis of silver nanoparticles [30]. Moreover, the results are in good agreement with those found in the literature [31].

It can be concluded from the FT-IR that some of the bioorganic compounds from *Citrus* sp. peel extracts such as flavonoids, alkaloids, coumarins, and phenolics may be involved in reducing the silver salt to Ag^0^. And these compounds act as capping and stabilizing agents which helps to prevent the silver nanoparticles from accumulation. However, the complete mechanism of the synthesis of silver nanoparticles in this reduction reaction by phytochemicals in the peel extracts of *Citrus* sp. fruits is to be further elucidated.

### 3.3. Transmission Electron Microscopy

**Figure 4:**
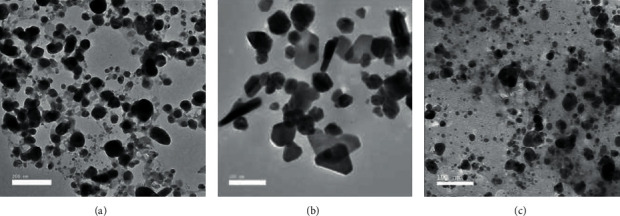
TEM image of silver nanoparticles synthesised using (a) lemon peel, (b) orange peel, and (c) green orange peel extract.

The morphological studies of synthesised silver nanoparticles were performed using transmission electron microscopy (TEM). TEM images of synthesised silver nanoparticles using lemon peel, orange peel, and green orange peel are shown in Figure 4(a)–4(c), respectively. Figure 4(a) reveals that the particles are well dispersed and spherical in shape, while some particles are found to be oval and/or having anisotropic structures of irregular shapes. Such variation in size and shape of biosynthesized nanoparticles is common [32]. TEM analysis showed that most particles had a size of about 10–70 nm.  Figure 4(b) shows the TEM images of silver nanoparticles synthesised using orange peel extract which has a mixed population of silver nanoparticles containing triangular, rod, near spherical, spherical, and hexagonal shapes with the sizes range from 5 to 80 nm. There are some small sized particles which might have been in the growing stage.  Figure 4(c) shows silver nanoparticles synthesised using green orange peel extract having shapes mostly spherical and irregular with the size range of 10–50 nm.

Citrus fruits contain natural antioxidants, such as, flavonoids, alkaloids, coumarins and phenolics, and their peels, which represent a primary waste fraction, are used as sources of molasses, pectin, cold-pressed oils, and limonene [33]. The rich source of citric acid and ascorbic acid in the citrus fruit extract may possibly responsible for reduction of metal ions and efficient stabilization of synthesised nanoparticles. Since citrus fruits are rich source of citric acid, *Citrus limon*, *Citrus sinensis*, and *Citrus tangerina* were used as a bioreductant for the synthesis of silver nanoparticles.

Phenolic acids contain their phenolic nucleus and a carboxylic group form a resonance-stabilized phenoxy radical which accounts for their antioxidant potential. It is generally believed that the oxidation of phenols leads to stable phenoxy radical formation in the initial stage. This radical is extremely stabilized by delocalization across the whole molecule [34]. It is thus possible that the phenolic acids act as a reducing agent and are oxidized by silver nitrate, resulting in the formation of silver nanoparticles.

### 3.4. Antibacterial Effectiveness of Silver Nanoparticles

**Figure 5:**
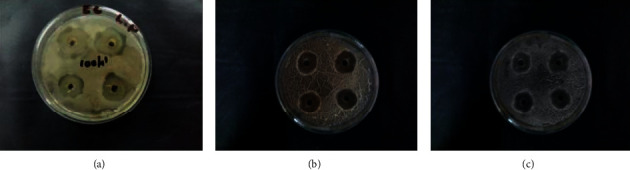
Antibacterial activity of silver nanoparticles synthesised using (a) lemon peel, (b) orange peel, and (c) green orange peel extract against *Escherichia coli*.

**Figure 6:**
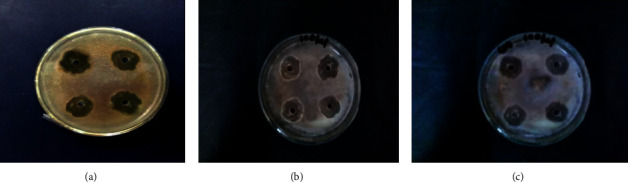
Antibacterial activity of silver nanoparticles synthesised using (a) lemon peel, (b) orange peel, and (c) green orange peel extract against *Staphylococcus aureus*.

The antibacterial activity of the silver nanoparticles synthesised by this method was studied by the well-diffusion method against the Gram-negative bacteria *Escherichia coli* and the Gram-positive bacteria *Staphylococcus aureus*.  Figure 5 and 6 show that the antibacterial activity of AgNPs synthesised using lemon peel (Figures 5(a) and 6(a)), orange peel (Figures 5(a) and 6(b)), and green orange peel (Figures 5(a) and 6(c)) against *Escherichia coli* and *Staphylococcus aureus*, respectively.

The antibacterial activities of AgNO_3_, plain lemon peel, orange peel, and green orange peel were also tested. The antibacterial effectiveness of the standard antibiotics such as Bactrim (*Escherichia coli*) and Cephalexin (*Staphylococcus aureus*) also checked. The values of zone of inhibition observed around the wells of synthesised silver nanoparticles are given in Table 1, and plain peel extracts (control) and the standard antibiotics are given in Table 2.

The results show that there is no specific antibacterial activity against *Escherichia coli* and *Staphylococcus aureus* by AgNO_3_ (Table 2). This may be due to its very low concentration (1 mM). The same amount of lemon peel, orange peel, and green orange peel extracts showed negligible amount of antibacterial activity when compared with the activity of silver nanoparticles for the dose of 60 *μ*l (Table 1 and 2). It is to be presumed that the peel extract of citrus fruits was used to possess the antibacterial activities and must be reflected through greater inhibition zone.However, they alone show very low activity due to its medium of extraction as well as lower concentration during experimentation.

It has been observed that silver nanoparticles were more effective against *Escherichia coli* than *Staphylococcus aureus*. Moreover, the bactericidal activity of silver nanoparticles was found to increase with increasing dosage (Table 1). Silver nanoparticles synthesised using orange peel extract showed more inhibition than the others (Table 1).This may be due to various shapes and sizes of nanoparticles. The literature reveals that the bactericidal property of silver nanoparticles entirely depends on the particle size and dose and is more active against Gram-negative bacteria than Gram-positive bacteria [35]. The possible reason might be the difference in the composition and thickness of the peptidoglycan layer in the cell wall. Gram-positive bacteria possess a three-dimensional peptidoglycan layer of ∼80 nm (10 times thicker than Gram-negative bacteria) and are less susceptible to attack by silver nanoparticles. It is said that silver interacts with thiol groups of protein on cell membrane, which results into blocking of respiration and producing ultimate death [36].

It has also been suggested that interaction of silver nanoparticles with cell wall increases the membrane permeability by forming pores or pits and thereby causing death of bacteria [37].

## 4. Conclusions

In this study, a simple approach was used to synthesise AgNPs using the peels of three citrus fruits. The phytochemicals present in the peel extracts act as an effective reducing and stabilizing agents. The synthesised nanoparticles were characterised using UV-visible spectroscopy, FT-IR spectroscopy, and TEM . The TEM analysis discovered that the biologically synthesised silver nanoparticles were well dispersed with no agglomeration. The size of nanoparticles ranges from 5 to 80 nm with different shapes such as spherical, triangular, hexagonal, and rod. This AgNPs showed a broad-spectrum antibacterial activity against Gram-negative (*Escherichia coli*) and Gram-positive (*Staphylococcus aureus*) bacteria. The antibacterial activity of silver nanoparticles was well demonstrated by the zone of inhibition. This method of synthesising the AgNPs is cost-effective and eco-friendly.

## Figures and Tables

**Table 1:** Effect of silver nanoparticles on the growth of bacterial species.

Dose of silver nanoparticles (*μ*l)	Zone of inhibition (mm)					
	Lemon peel		Orange peel		Green orange peel	
	*E. coli*	*S. aureus*	*E. coli*	*S. aureus*	*E. coli*	*S. aureus*
20	14.16 ± 0.76	8.1 ± 0.28	16.16 ± 0.76	9.17 ± 0.28	11.83 ± 1.60	7.17 ± 0.76
40	16.16 ± 0.76	9.8 ± 0.76	17.83 ± 0.76	11.67 ± 0.76	15.5 ± 0.5	9.67 ± 1.26
60	17.83 ± 0.76	13 ± 0.5	18.83 ± 0.76	12.83 ± 0.76	16.33 ± 1.26	11.33 ± 0.76
80	19.50 ± 0.5	14.66 ± 0.57	20.50 ± 0.5	15.83 ± 0.76	19.5 ± 0.5	13.16 ± 0.76
100	20.16 ± 1.04	16 ± 0.5	21.33 ± 0.28	17.16 ± 0.76	19.83 ± 1.76	14.17 ± 0.76

All the values added here are mean values with ± standard deviation.

**Table 2:** Effect of plain peel extracts (control) and effect of standard antibiotics on the growth of bacterial species.

Dose of solution (*μ*l)	Zone of Inhibition (mm)									
	Plain lemon peel		Plain orange peel		Plain G. orange peel		AgNO_3_		Bactrim	Cephalexin
	*E. coli*	*S. aureus*	*E. coli*	*S. aureus*	*E. coli*	*S. aureus*	*E. coli*	*S. aureus*	*E. coli*	*S. aureus*
60	7.83 ± 0.76	7 ± 0.5	8.16 ± 07.6	5.83 ± 0.76	7.5 ± 0.5	-	-		15.16 ± 1.04	9.83 ± 1.26

All the values added here are mean values with  ± standard deviation.

## Data Availability

The data used to support the findings of this study are available from the corresponding author upon request.
